# Role of circulating angiogenin levels in portal hypertension and TIPS

**DOI:** 10.1371/journal.pone.0256473

**Published:** 2021-08-25

**Authors:** Alexander Queck, Frank E. Uschner, Philip G. Ferstl, Martin Schulz, Maximilian J. Brol, Michael Praktiknjo, Robert Schierwagen, Sabine Klein, Christian P. Strassburg, Carsten Meyer, Christian Jansen, Marie-Luise Berres, Jonel Trebicka

**Affiliations:** 1 Department of Internal Medicine 1, University Hospital, Johann Wolfgang Goethe-University, Frankfurt am Main, Germany; 2 Department of Internal Medicine I, University Hospital Bonn, University of Bonn, Bonn, Germany; 3 Department of Radiology, University Hospital, University Bonn, Bonn, Germany; 4 Department of Internal Medicine III, RWTH Aachen, Aachen, Germany; 5 European Foundation for the Study of Chronic Liver Failure, Barcelona, Spain; Medizinische Fakultat der RWTH Aachen, GERMANY

## Abstract

**Background:**

Pathogenesis of portal hypertension is multifactorial and includes pathologic intrahepatic angiogenesis, whereby TIPS insertion is an effective therapy of portal hypertension associated complications. While angiogenin is a potent contributor to angiogenesis in general, little is known about its impact on TIPS function over time.

**Methods:**

In a total of 118 samples from 47 patients, angiogenin concentrations were measured in portal and inferior caval vein plasma at TIPS insertion (each blood compartment n = 23) or angiographic intervention after TIPS (each blood compartment n = 36) and its relationship with patient outcome was investigated.

**Results:**

Angiogenin levels in the inferior caval vein were significantly higher compared to the portal vein (P = 0.048). Ten to 14 days after TIPS, inferior caval vein angiogenin level correlated inversely with the portal systemic pressure gradient (P<0.001), measured invasively during control angiography. Moreover, patients with TIPS revision during this angiography, showed significantly lower angiogenin level in the inferior caval vein compared to patients without TIPS dysfunction (P = 0.01).

**Conclusion:**

In cirrhosis patients with complications of severe portal hypertension, circulating levels of angiogenin are derived from the injured liver. Moreover, angiogenin levels in the inferior caval vein after TIPS may predict TIPS dysfunction.

## Introduction

Pathogenesis of portal hypertension (PH) in liver cirrhosis is multifactorial and orchestrated by an increase of sinusoidal resistance due to liver fibrosis [1], endothelial dysfunction [2], bacterial translocation [3] and rise of portal venous blood inflow secondary to splanchnic vasodilatation [4]. In addition, pathological intra- and extra-hepatic angiogenesis contributes to PH [5–7].

The severity of PH is related to disease progression and causes complications, such as ascites, bleeding from gastro-esophageal varices and renal dysfunction [8]. For treatment of PH associated complications, transjugular intrahepatic porto-systemic shunt (TIPS) insertion reduces the portal-hepatic pressure gradient (PHPG) and improves patients’ outcome [9,10]. Nevertheless, TIPS dysfunction has been a frequent complication, especially in the era of uncovered bare metal stents [11]. Usually, vascular stent insertions provoke vascular healing and remodeling cascades [12], which eventually result in reendothelialization of the vein graft [13]. However, a pathological vascular response can lead to the development of pseudo-intimal hyperplasia and thrombosis, both of which are mechanisms of TIPS dysfunction [14,15].

While angiogenesis involves several messengers like vascular endothelial-derived growth factor (VEGF) [16], one essential mediator is angiogenin (also referred as ribonuclease 5), a protein constituted of 123 amino acids and a member of the ribonuclease superfamily [17,18]. Angiogenin stimulates blood vessels growth via interaction with endothelial and smooth muscle cells [19]. To enable this process, angiogenin provides ribonucleolytic activity [20,21] and the ability for nuclear translocation with enhancement of ribosomal RNA transcription in endothelial cells [22]. Moreover, angiogenin also induces signaling transduction and basement membrane degradation [23,24]. Besides angiogenesis, angiogenin also has anti-microbial and anti-inflammatory properties and seems to be essential for the maintenance of gut microbe homeostasis [25,26].

Therefore, we hypothesized that angiogenin is involved in and essential for (i) post-TIPS vascular healing and neo-vascularization processes and (ii) the anti-inflammatory response.

## Patients and methods

### Study oversight

This study presents portal and inferior caval vein angiogenin levels of patients undergoing TIPS insertion, as well as of patients during control angiography after TIPS. Patients were recruited between September 1998 and August 2003 at the Department of Internal Medicine I, University of Bonn, Germany. The study protocol was approved by the local ethics committee of the University of Bonn (029/13). Written informed consent was obtained from all patients before enrolment and patients agreed to all procedures as declared in the study protocol. All authors had access to the study data and reviewed and approved the final manuscript.

### Patients

A total of 118 samples from 47 patients with diagnosed liver cirrhosis and complications of PH was included in this study. For this purpose, angiogenin concentrations were measured in portal and inferior caval vein plasma at TIPS-insertion (each blood compartment samples n = 23) or angiographic intervention after TIPS (each blood compartment samples n = 36) (S1 Fig). Indications for TIPS insertion were secondary prophylaxis of variceal bleeding (n = 8; 35%), refractory ascites (n = 13; 56%) and hepatorenal syndrome (n = 2; 9%). Patients older than 18 years with clinical signs of liver cirrhosis and a multidisciplinary defined indication for TIPS insertion were included in our trial. Exclusion criteria were the presence of systemic infection, severe hepatic encephalopathy of unknown reason, severe hyperbilirubinemia, pulmonary hypertension, or pregnancy. After a mean period of 14 days after TIPS insertion, 39 patients received control angiography as a routine procedure, as previously described [27].

### Study design

After study inclusion, patients received TIPS insertion or angiographic intervention after TIPS as recommended by an interdisciplinary expert team. TIPS (8–10 mm Wallstent, Boston Scientific, MA, USA) insertion was performed as previously described [28]. During the procedures (TIPS or angiographic intervention after TIPS), portal and hepatic venous pressures were invasively measured with a pressure transducer system (Combitrans, Braun, Melsungen, Germany) and a multichannel monitor (Sirecust, Siemens, Germany). Per definition, the difference between the portal and hepatic venous pressure was defined as PHPG. After cannulating the right branch of the portal vein, we harvested blood from the portal and the inferior caval vein (at the level of the hepatic veins) in EDTA tubes to obtain material for angiogenin analysis. If invasive flow criteria for TIPS dysfunction (shunt stenosis>50%, occlusion or flow reduction) were fulfilled during control angiography, TIPS revision was performed. All TIPS insertions or angiographic interventions after TIPS were performed without general anesthesia. After collection of the patient’s blood, we centrifuged the samples at 3000 revolutions per minute for 15 minutes at 4°C. Next, plasma samples were stored at -80°C. Biochemical parameters were analyzed using standard methods.

### Measurement of angiogenin concentrations

Plasma concentrations of angiogenin were assessed with a cytometric bead array (Becton Dickinson, Heidelberg, Germany) according to the manufacturer’s instructions, quantified in undiluted samples in duplicates.

### Statistical analyses

GraphPad Prism 9.1.2 (GraphPad Software, Inc.) and BIAS® (version 11.08) for Windows were used for the performance of statistical analysis. To check for normal distribution, the D’Agostino and Pearson omnibus normality test was performed. Parametric (unpaired T-test) or nonparametric (Mann-Whitney) tests were applied accordingly. For analyses of paired observations, such as angiogenin progression over time, the paired T-test (parametric) or the Wilcoxon matched-pairs signed rank test (non-parametric) were used. For analysis of correlations, the nonparametric Spearmen’s or the parametric Pearson’s correlation were used. P values <0.05 were defined as statistically significant.

## Results

### Angiogenin levels are higher in the inferior caval vein compared to the portal vein

**Fig 1:**
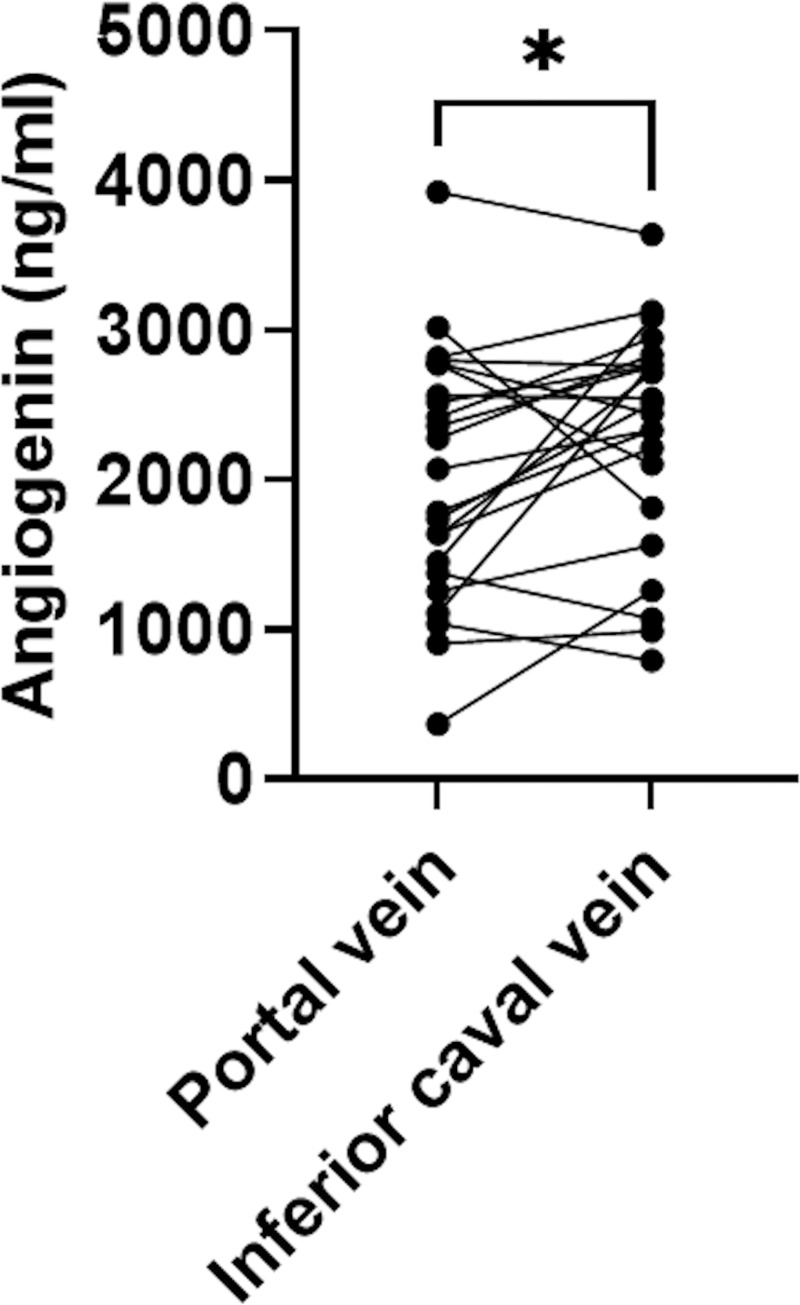
Portal and inferior caval vein levels of angiogenin at TIPS insertion. For statistical analysis, the paired t-test was used (P = 0.048) and presented as scatter plot. TIPS: Transjugular intrahepatic portosystemic shunt. *n = 23*.

Angiogenin level were measured in the portal and the inferior caval vein blood of patients with decompensated cirrhosis at TIPS insertion (each compartment: n = 23). At the time of TIPS insertion, covert hepatic encephalopathy was present in four patients (17%), and 65% of the patients presented with refractory ascites. Mean MELD score at TIPS insertion was 14 and most of the patients were classified as Child-Pugh B (74%), with a mean score of 8. Patients presented with mean low platelet counts (122/μL), and hypoalbuminemia (3.1 g/dL). Mean PHPG was 21 mmHg at, and 9 mmHg after TIPS insertion. As expected after TIPS creatinine levels decreased, while platelet counts and gamma-glutamyltransferase levels increased (S1 Table). Mean portal vein angiogenin levels were significantly lower compared to the levels in the inferior caval vein (portal vein: 2027 ng/mL; inferior caval vein: 2321 ng/mL; P = 0.048) (Table 1) (Fig 1). We observed neither a significant difference, nor a correlation between angiogenin concentrations and the year of blood sampling (S2 Fig).

**Table 1:** Characteristics of patients at TIPS insertion.

Parameter	TIPS insertion (n = 23)
Mean (standard deviation) or absolute (percentage)	
**General**	
Age (years)	61 (9)
Indication for TIPS insertion	
• *bleeding/ascites/bleeding and ascites/HRS*	6/13/2/2 (26/56/9/9)
MELD score	14 (7)
Child-Pugh score	8.0 (1.2)
Child-Pugh class (A/B/C)	4/17/2 (17/74/9)
**Clinical events**	
Hepatic encephalopathy (no/yes)	19/4 (83/17)
Ascites (Stage 0/1/2)	3/5/15 (13/22/65)
Varices (Stage 0/1/2/3)	2/6/11/4 (9/26/48/17)
**Laboratory**	
Sodium (mmol/L)	135 (3.4)
GPT (U/I)	24 (14)
GGT (U/I)	87 (108)
Creatinine (mg/dL)	1.9 (1.2)
Bilirubin (mg/dL)	2.0 (3.3)
WBC (10^3^/μL)	6.0 (4.1)
Albumin (g/dL)	3.1 (0.8)
INR	1.2 (0.2)
Platelets (/μL)	122 (60)
**Angiogenin**	
Portal vein (ng/mL)	2027 (836)
Inferior caval vein (ng/mL)	2321 (749)

GGT: Gamma-glutamyltransferase; GPT: Glutamate pyruvate transaminase; HRS: Hepatorenal syndrome; INR: International normalized ratio; MELD: Model for end-stage liver disease; TIPS: Transjugular intrahepatic portosystemic stent shunt; WBC: White blood cell count.

### Angiogenin concentrations in the inferior caval vein are inversely correlated with PHPG after TIPS

**Fig 2:**
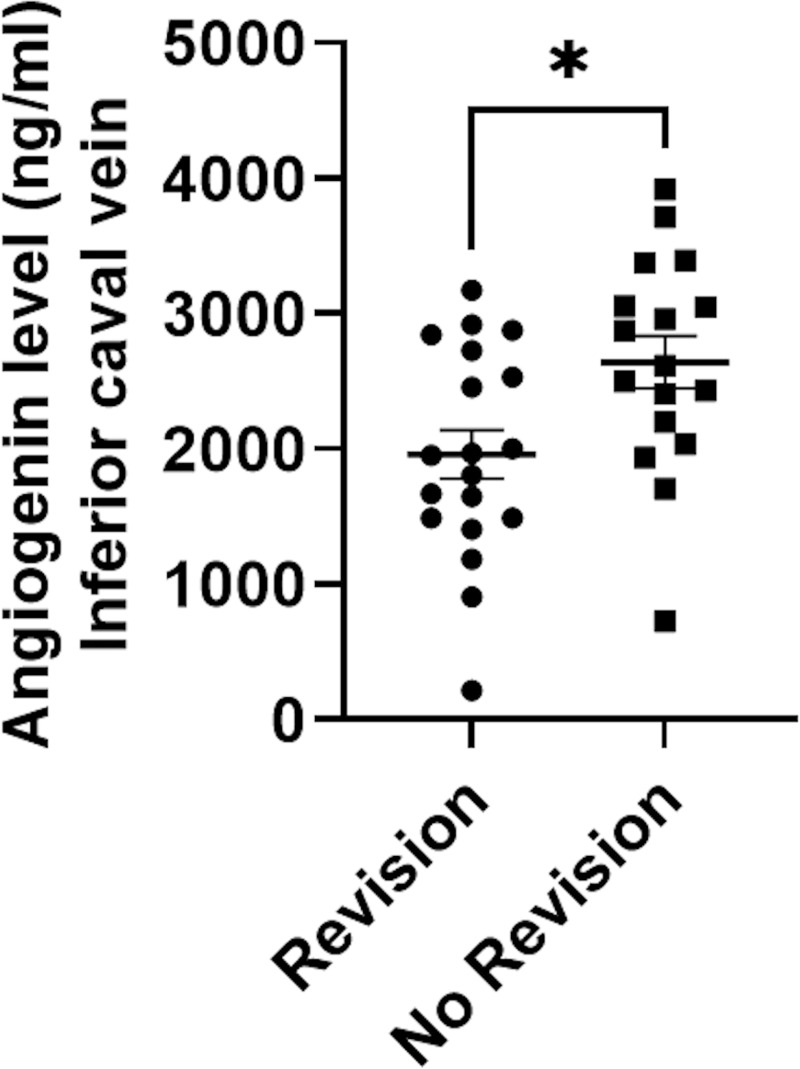
Angiogenin levels in the inferior caval vein during follow-up angiography. Differences between inferior caval vein angiogenin levels in dependency of the need for TIPS revision at control angiography (P = 0.01). For statistical analysis, the unpaired t-test was used and presented as scatter plot with mean and standard error of the mean. TIPS: Transjugular intrahepatic portosystemic shunt. n = 36.

**Fig 3:**
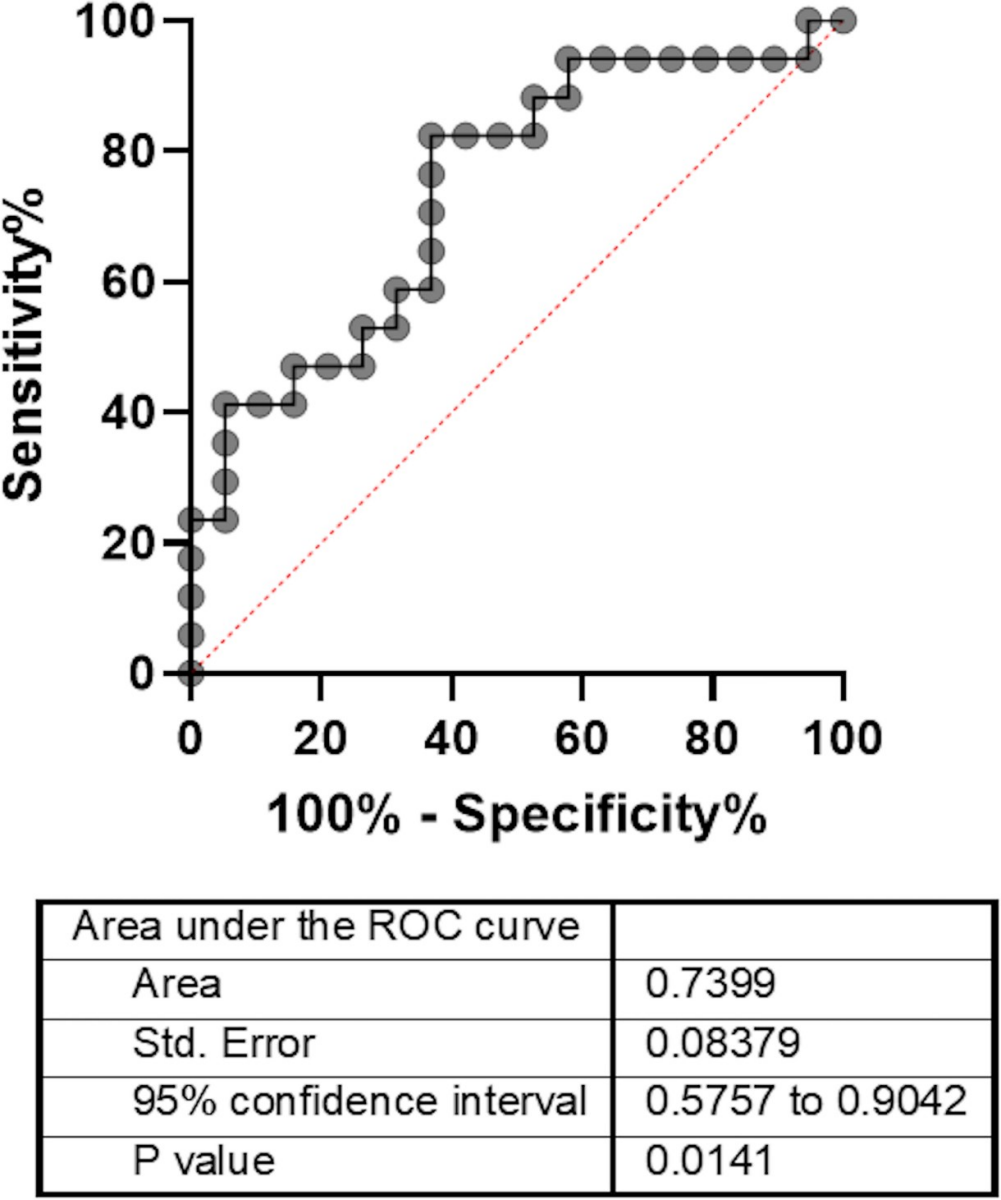
AUROC of angiogenin for TIPS dysfunction. The area under the ROC curve of angiogenin for TIPS dysfunction was 0.74 (0.6–0.9); P = 0.01. TIPS: Transjugular intrahepatic portosystemic shunt. n = 36.

**Fig 4:**
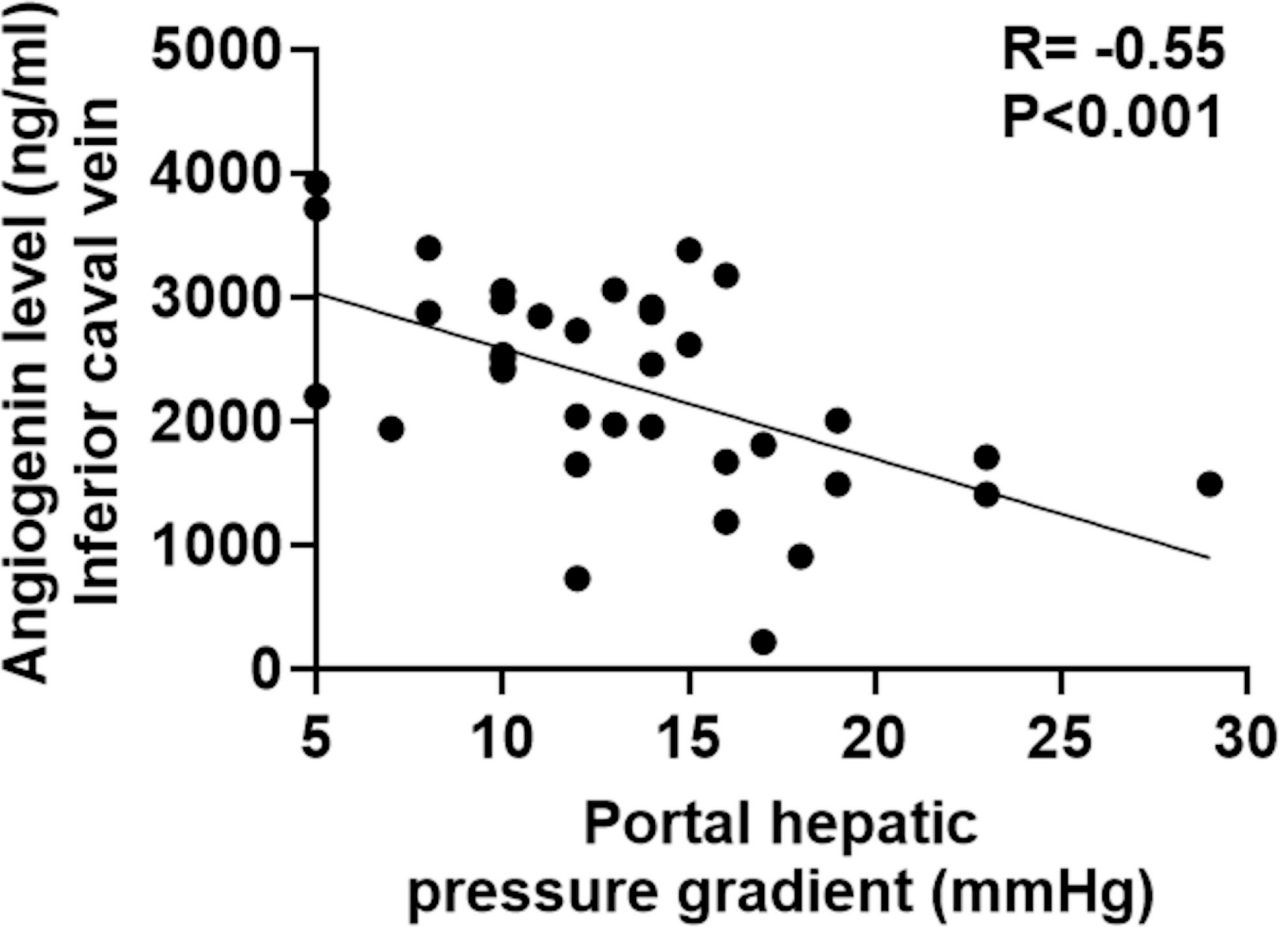
Correlation between angiogenin level in the inferior caval vein at follow-up angiography and portal hepatic pressure gradient. Pearson’s correlation test was used for analysis of correlations between portal hepatic pressure gradient and inferior caval vein angiogenin levels at follow up angiography. TIPS: Transjugular intrahepatic portosystemic shunt. _*n*_ = 36.

Angiogenin levels were measured in 72 samples of 39 patients in portal and inferior caval vein plasma (each compartment: n = 36) of 39 patients receiving control angiography after TIPS insertion. At this timepoint, mean MELD score was 10, while hypoalbuminemia (3.0 g/dL), low platelet count (125/μL) and increased GGT levels (151 U/I) were observed as surrogate markers of advanced liver disease and PH. Mean angiogenin levels in the portal and the inferior caval vein were not significantly different (portal vein: 2014 ng/mL and inferior caval vein: 2282ng/mL; P = 0.4) (Table 2). Twenty-two of the 39 patients (56%) received TIPS revision due to TIPS dysfunction, defined by radiological flow criteria at control angiography, whereby MELD score and other general laboratory parameters were not significantly different between patients with and without TIPS dysfunction. Nevertheless, patients with dysfunction showed significantly lower angiogenin levels in the inferior caval vein compared to patients without (1961 ng/mL versus 2641 ng/mL; P = 0.014) (Table 2) (Fig 2). In these cases, the area under the ROC curve of angiogenin for TIPS dysfunction was 0.74 (0.6–0.9, P = 0.01) (Fig 3). Moreover, inferior caval vein angiogenin levels were inversely correlated with PHPG at control angiography in all patients, regardless of TIPS function (R = -0.55; P<0.001) (Fig 4).

**Table 2:** Characteristics of patients at follow-up angiography.

Parameter	Follow-up angiography			
	All (n = 39)	Revision (n = 22/56%)	No revision (n = 17/44%)	P-value
Mean (standard deviation) or absolute (percentage)				
Age (years)	58 (8.0)	57 (7.0)	59 (8.0)	0.4
MELD score	10 (5)	10 (5.0)	10 (5.0)	0.8
Sodium (mmol/L)	137 (4.2)	137 (4.6)	138 (3.4)	0.4
GPT (U/I)	33 (27)	27 (18)	41 (34)	0.3
GGT (U/I)	151 (105)	158 (108)	143 (103)	0.6
Creatinine (mg/dL)	1.4 (1.3)	1.4 (1.1)	1.5 (1.6)	0.6
Bilirubin (mg/dL)	1.6 (1.2)	1.5 (1.4)	1.6 (0.9)	0.2
WBC (10^3^/μL)	5.9 (2.3)	5.5 (2.4)	6.5 (2.1)	0.1
Albumin (g/dL)	3.0 (0.6)	3.1 (0.6)	2.9 (0.6)	0.5
INR	1.2 (0.2)	1.2 (0.2)	1.2 (0.2)	1.0
Platelets (/μL)	125 (58)	127 (64)	121 (53)	0.8
**Angiogenin**				
Portal vein (ng/mL)	2014 (1021)	1889 (994)	2405 (1014)	0.14
Inferior caval vein (ng/mL)	2282 (848)	1961 (778)	2641 (795)	**0.014**

GGT: Gamma-glutamyltransferase; GPT: Glutamate pyruvate transaminase; INR: International normalized ratio; MELD: Model for end-stage liver disease; WBC: White blood cell count.

### Angiogenin correlates with the level of systemic inflammation at control angiography after TIPS

**Fig 5:**
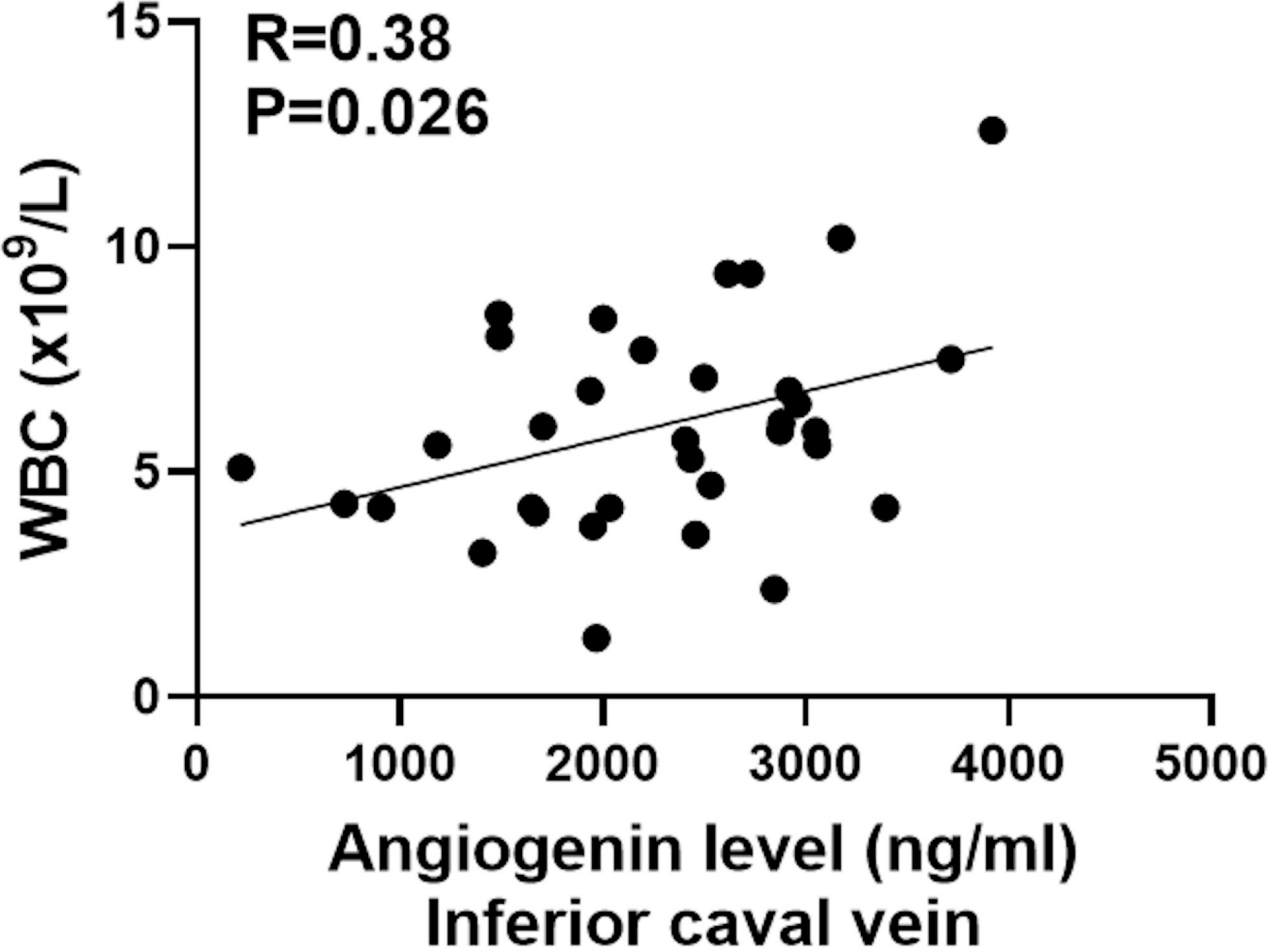
Correlation between inferior caval vein angiogenin concentrations and systemic white blood cell count at control angiography. Pearson’s correlation test was used for analysis of correlations between systemic WBC and inferior caval vein angiogenin level during control angiography. WBC: White blood cell count. *n* = 34.

While systemic white blood cell count and angiogenin levels in the inferior caval vein during TIPS insertion did not correlate (R = 0.02, P = 0.36), significant correlations between systemic white blood cell count and angiogenin concentrations in the inferior caval vein were observed at control angiography after TIPS (R = 0.38, P = 0.026) (Fig 5).

## Discussion

This study demonstrates that the injured liver is the major source of angiogenin in patients with complications of PH receiving TIPS. Furthermore, inferior caval vein angiogenin concentrations were correlated with PHPG after TIPS insertion. Hence, inferior caval vein angiogenin levels may predict TIPS dysfunction.

This study analyzed the source of angiogenin. Since higher angiogenin concentrations were measured in the inferior caval vein than in the portal vein, the main source of angiogenin in decompensated cirrhosis may be the diseased liver. While decompensation of cirrhosis leads to upregulation of pro-inflammatory [29] as well as pro-angiogenic signaling [30], both mechanisms could trigger hepatic angiogenin expression, possibly because PH is due to vascular remodeling and endothelial dysfunction associated with—and caused by dysregulated angiogenesis [31]. Nevertheless, the inferior caval vein angiogenin concentrations did not correlate with the level of PHPG at TIPS insertion. This strong inverse correlation between inferior caval vein angiogenin levels and PHPG only occurred during control angiography, 14 days after TIPS. Interestingly, lower angiogenin concentrations in the inferior caval vein were measured in patients with TIPS dysfunction. Therefore, one may hypothesize that an acute event, such as TIPS insertion itself and/or in-stent thrombosis influences the angiogenin expression. In line with this hypothesis, *in-vivo* studies of angiogenin during angiogenic and remodeling processes, such as placental growth in pregnancy, reported insufficient angiogenin concentrations to be associated with pathological blood flow [32]. Therefore, one explanation for the relationship between angiogenin levels and post-TIPS PHPG may be a pathological vascular response with consecutive inaccurate reendothelialization of TIPS and/or inadequate remodeling (healing) of the hepatic vascular bed. Thus, this pathologic response in post-TIPS angiogenesis signaling could increase the risk of pseudo-intimal hyperplasia and/or TIPS thrombosis, leading to TIPS dysfunction. This possibly explains why lower angiogenin levels were associated with TIPS dysfunction.

Another—and maybe synergetic—effect could be flow dependency of angiogenin. Patients with TIPS dysfunction and, therefore, reduced sinusoidal perfusion may have a lower hepatic angiogenin release compared to patients with adequate TIPS perfusion.

Moreover, angiogenin seems to be regulated as an acute phase protein [33], probably because of the reported involvement in the innate immunity [34]. Along these lines, previous studies have demonstrated that interleukin 6 (IL-6), a pro-inflammatory mediator, induced synthesis and secretion of angiogenin in a human hepatic (HEPG2) cell line [35]. Of note, bacterial translocation in decompensated cirrhosis is also known to generate high IL-6 concentrations and is associated with acute-on-chronic liver failure (ACLF) [36] and platelet activation [37]. In our patients this correlation between angiogenin and inflammation was also observed. High post-TIPS angiogenin levels in the inferior caval vein correlated with high white blood cell counts. Since higher angiogenin concentrations also correlated with better TIPS function, it may be hypothesized that post-TIPS angiogenin secretion, influenced by pro-inflammatory signaling, is also protecting the hepatic vascular system against this pro-inflammatory damage. This is nicely paralleled by the fact that angiogenin is a known initiator of the stress response process in mammalian cells [38] and plays a protective role in hypoxic-induced cellular damage [39,40]. Moreover, this bacterial translocation and inflammation may also have a strong effect on platelet aggregation in decompensated cirrhosis [37], and could be related to TIPS dysfunction. The anti-pathogenic and anti-inflammatory capabilities of angiogenin may have anti-thrombotic effects via reduced activation of platelet aggregation and therefore improve TIPS function.

There exist no reliable markers of TIPS dysfunction to date. Angiogenin may be useful for the non-invasive diagnosis of TIPS dysfunction, especially since ultrasound indications of TIPS dysfunction are not always conclusive.

This pilot study has several limitations. First, a control group of healthy individuals is missing. In addition, the sample size is small and monocentric, while a validation cohort for angiogenin is not available. Moreover, a change over time of angiogenin concentrations cannot be excluded. Nevertheless, no correlation between angiogenin levels and the timepoint of sampling was found. Also, a relationship between angiogenin and SPSS (spontaneous portosystemic shunts) cannot be excluded. Finally, although we sampled the blood from the inferior caval vein at the level of the hepatic veins, we cannot exclude venous admixture, originating from other organs.

In summary, post-TIPS angiogenin expression seems to be beneficial. Thus, angiogenin may play a relevant role not only in vascular healing processes, but also in the inflammatory response and coagulation conditions of the blood in the hepatic compartment. In conclusion, while angiogenin may be useful as a non-invasive marker of TIPS dysfunction, further validation is required.

## Supporting information

S1 Fig Number of patients at TIPS insertion and control angiography (A) and number of plasma samples in the portal and inferior caval vein at TIPS insertion and control angiography (B).
(TIF)

S2 Fig Angiogenin concentrations in dependency of the year of blood sampling A) 1998–2000 vs. 2001–2003 B) Correlation between angiogenin concentrations and the year of blood sampling. A) For statistical analysis, the unpaired t-test was used (P = 0.1) and presented as scatter plot. Samples: 1998–2000 n = 48 and 2001–2003 n = 70. B) For statistical analysis Pearson’s correlation test was used (P = 0.79). Samples n = 118.
(TIF)

S1 Table General characteristics of the subset of patients sampled at TIPS, as well as at the control angiography. GGT: Gamma-glutamyltransferase; GPT: Glutamate pyruvate transaminase; INR: International normalized ratio; MELD: Model for end-stage liver disease; WBC: White blood cell count.
(DOCX)
