# Supplementary figures and images for: Role of circulating angiogenin levels in portal hypertension and TIPS
Source: PLoS One. 2021 Aug 25;16(8):e0256473. doi: 10.1371/journal.pone.0256473 (PMC8386873; doi:10.1371/journal.pone.0256473)

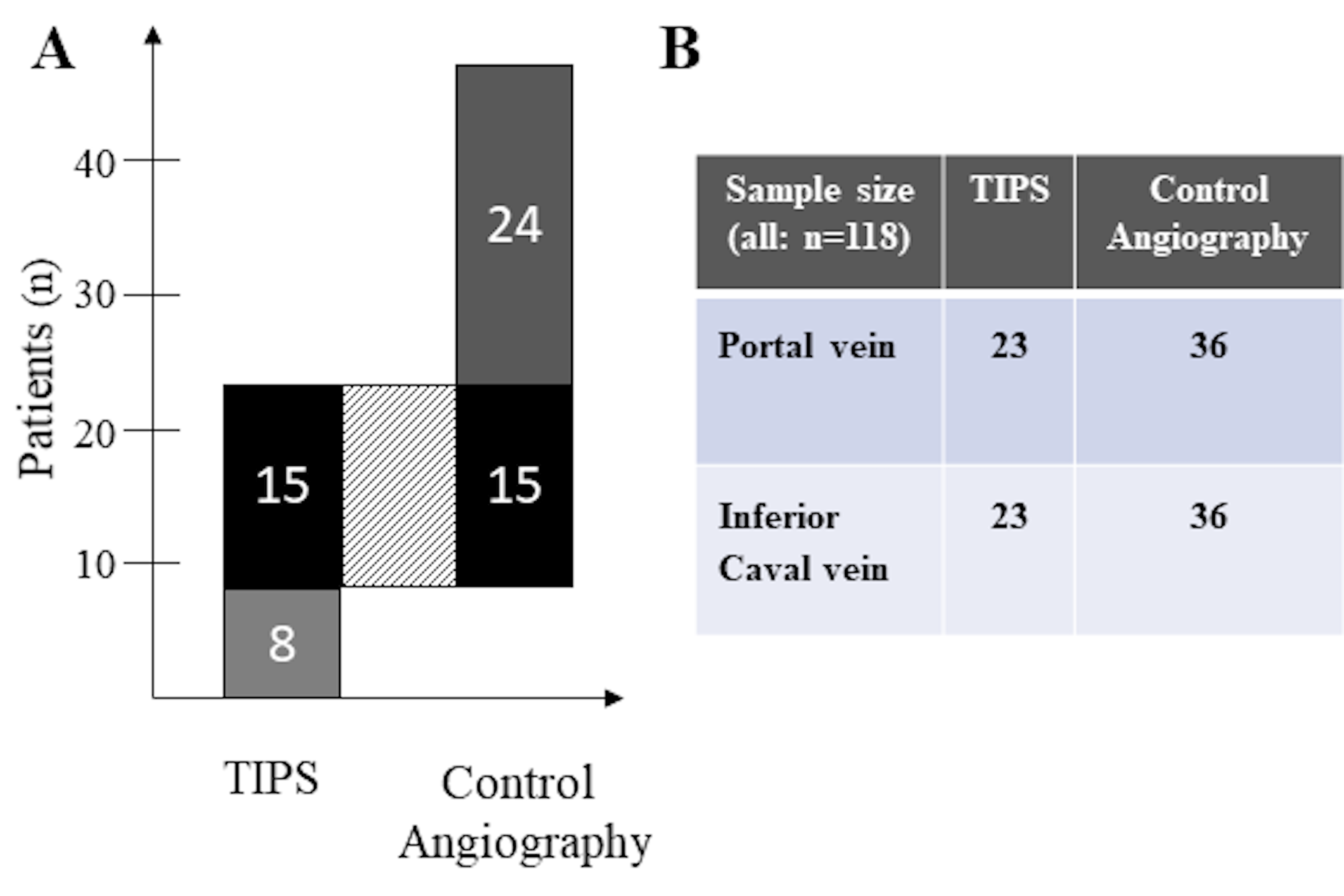

Supplement: S1 Fig — Number of patients at TIPS insertion and control angiography (A) and number of plasma samples in the portal and inferior caval vein at TIPS insertion and control angiography (B). (TIF) [file pone.0256473.s001.tif]

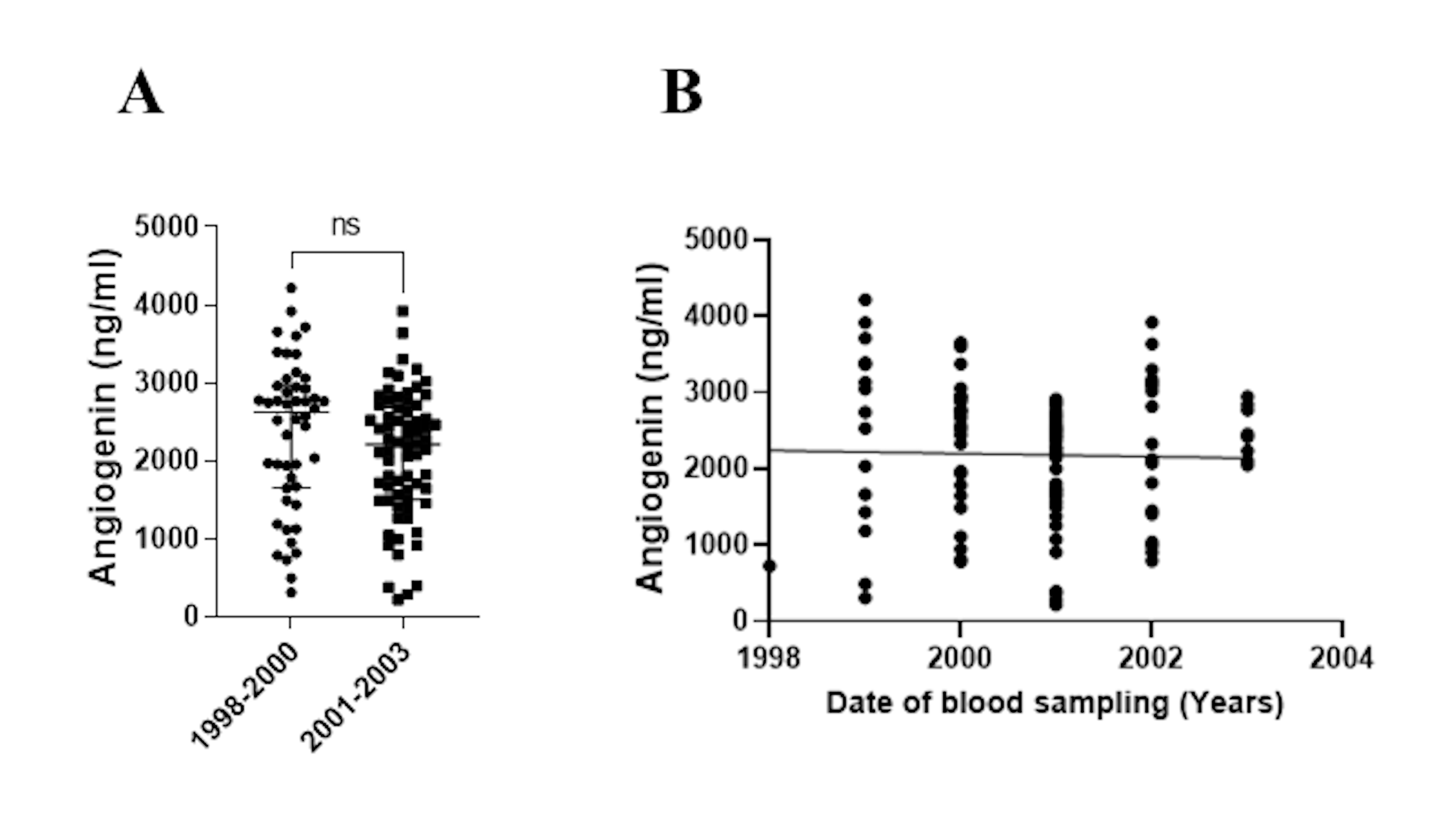

Supplement: S2 Fig — Angiogenin concentrations in dependency of the year of blood sampling A) 1998–2000 vs. 2001–2003 B) Correlation between angiogenin concentrations and the year of blood sampling. A) For statistical analysis, the unpaired t-test was used (P = 0.1) and presented as scatter plot. Samples: 1998–2000 n = 48 and 2001–2003 n = 70. B) For statistical analysis Pearson’s correlation test was used (P = 0.79). Samples n = 118. (TIF) [file pone.0256473.s002.tif]
