# Supplementary material for: Role of circulating angiogenin levels in portal hypertension and TIPS
Source: PLoS One. 2021 Aug 25;16(8):e0256473. doi: 10.1371/journal.pone.0256473 (PMC8386873; doi:10.1371/journal.pone.0256473)
Supplement: S1 Table — GGT: Gamma-glutamyltransferase; GPT: Glutamate pyruvate transaminase; INR: International normalized ratio; MELD: Model for end-stage liver disease; WBC: White blood cell count. (DOCX) [file pone.0256473.s003.docx]

| **Parameter** | **Patient characteristics** | | |
| --- | --- | --- | --- |
|  | **TIPS insertion**  **(N=15)** | **Control angiography (N=15)** | **P-value** |
| Mean (standard deviation) or absolute (percentage) | | |  |
| MELD score  Sodium (mmol/L)  GPT (U/I)  GGT (U/I)  Creatinine (mg/dL)  Bilirubin (mg/dL)  WBC (10^3^/µL) Albumin (g/dL)  INR  Platelets (/µL) | 13 (7.0)  135 (4.0)  23 (19)  98 (130)  1.9 (1.3)  1.3 (0.6)  5.5 (2.2)  3.3 (0.7)  1.2 (0.3)  120 (56) | 12 (6.0)  137 (3.0)  33 (26)  149 (94)  1.6 (1.3)  1.7 (1.1)  6.0 (2.2)  2.7 (0.6)  1.2 (0.2)  146 (46) | 0.6  0.08  0.08  0.02*  0.04*  0.06  0.25  0.5  0.09  0.007** |
